# Supplementary material for: Training instance segmentation neural network with synthetic datasets for crop seed phenotyping
Source: Commun Biol. 2020 Apr 15;3:173. doi: 10.1038/s42003-020-0905-5 (PMC7160130; doi:10.1038/s42003-020-0905-5)
Supplement: Supplementary file 1 — Supplementary Information [file 42003_2020_905_MOESM1_ESM.pdf]

Supplementary Figures

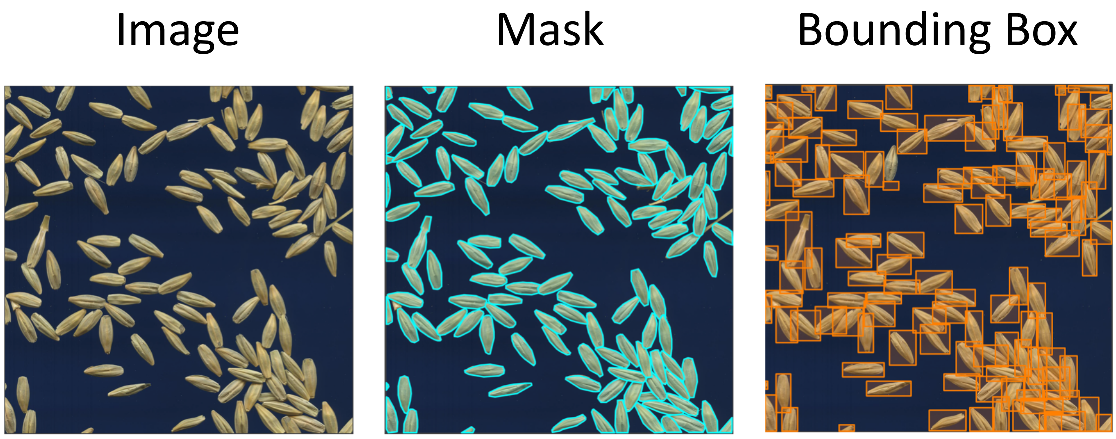

Supplementary Figure 1 | Example of a manual annotation for creating a training data. A bounding box coordinate and object mask regions are required for training an instance segmentation neural network, which are labor intensive process.

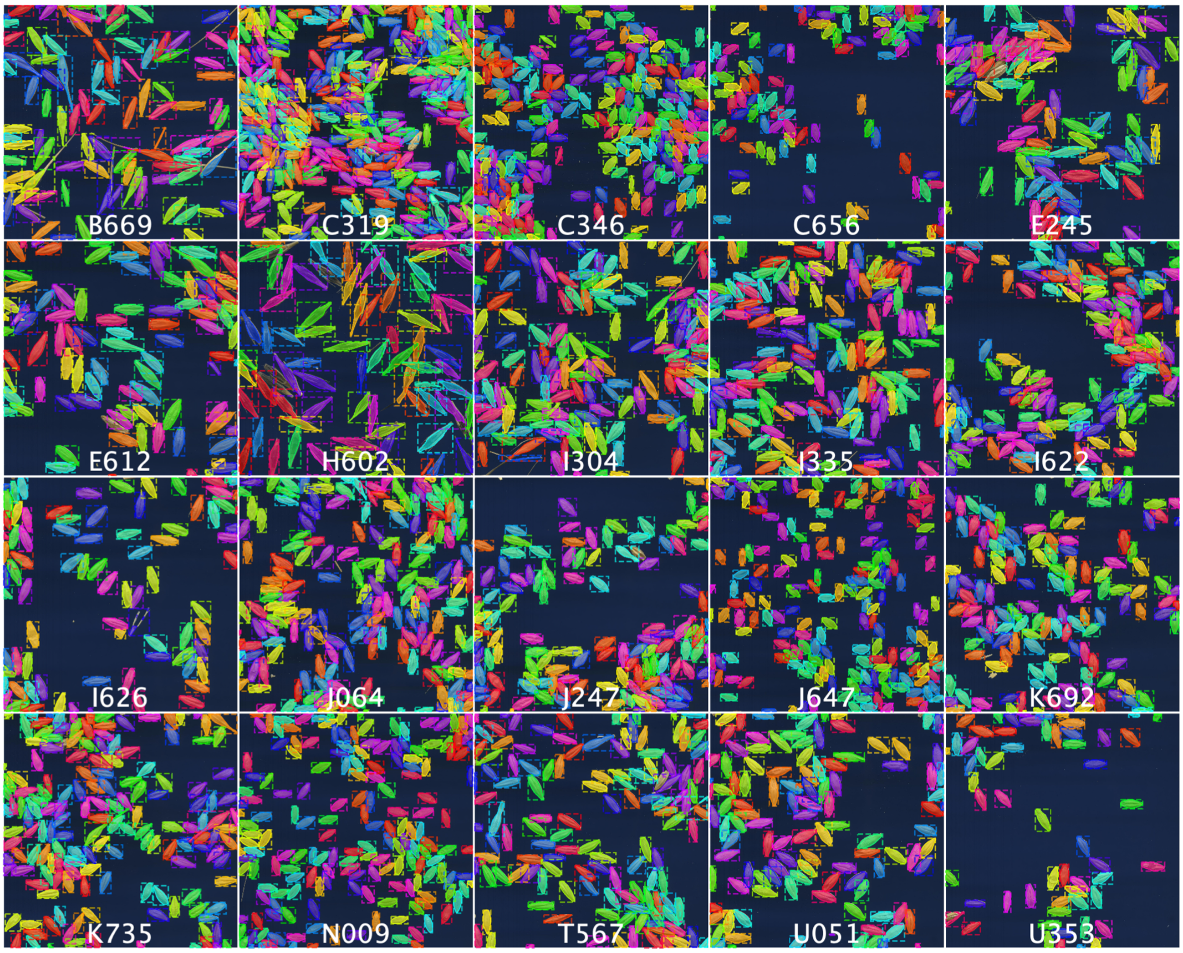

Supplementary Figure 2 | All graphical output of the real-world test data annotated by a trained Mask-RCNN.

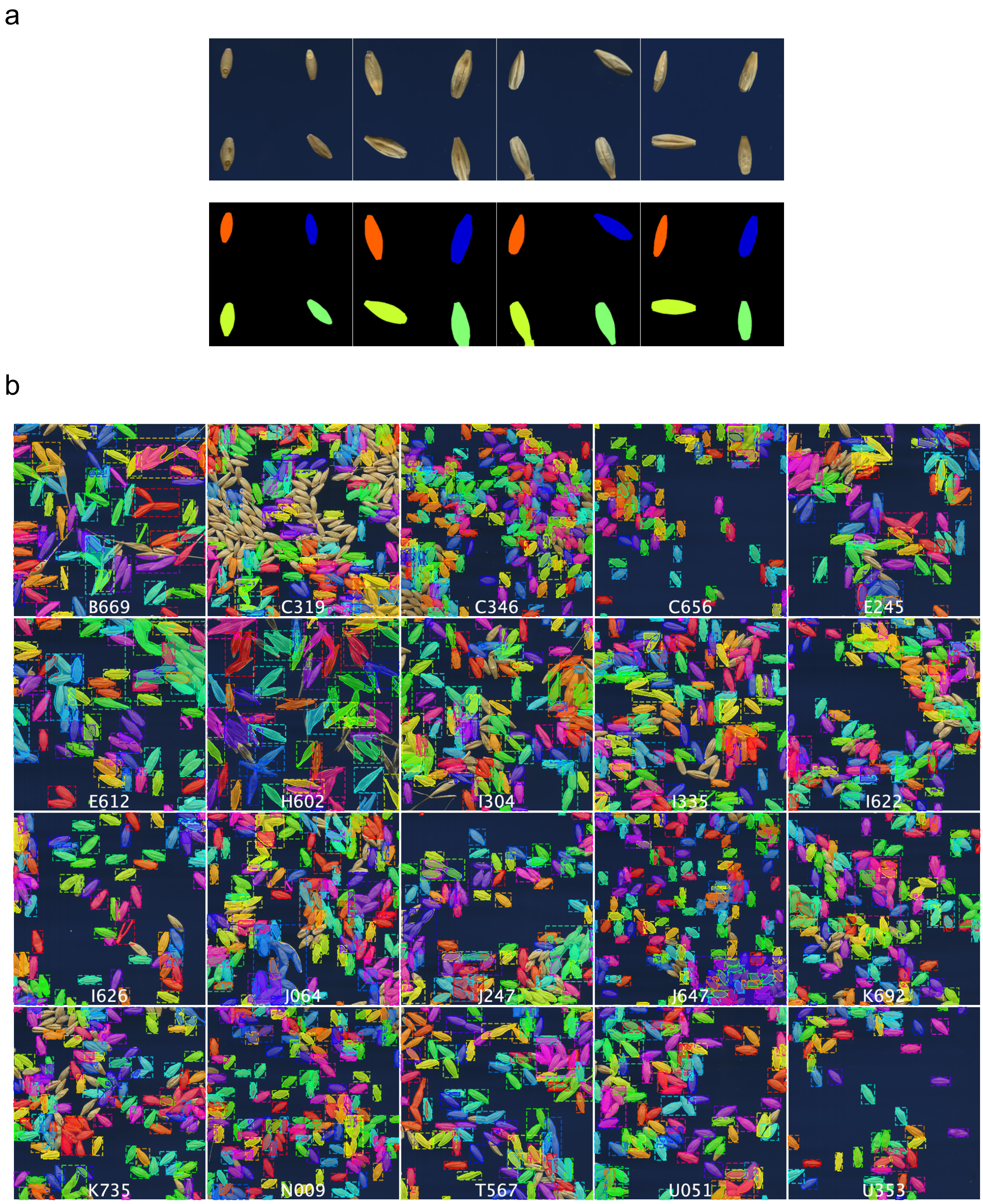

Supplementary Figure 3 | (a) The representative images and masks of the training dataset created from manually annotated 400 seeds without domain randomization. (b) All graphical output of the real-world test data annotated by a trained Mask-RCNN with the dataset shown in (a).

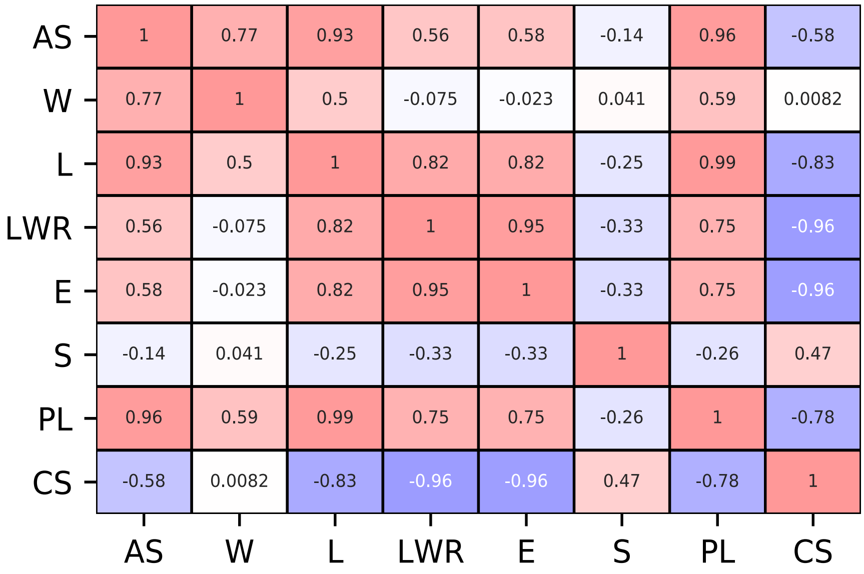

Supplementary Figure 4 | Pearson correlation coefficient matrix of 8 morphological descriptors.

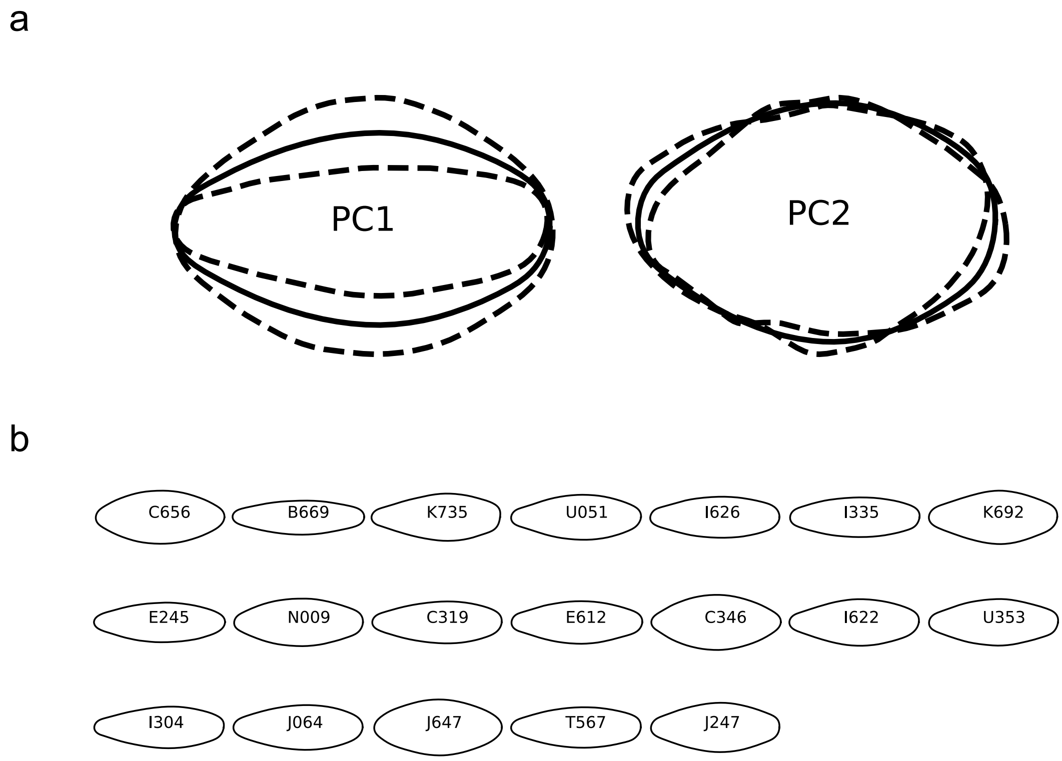

Supplementary Figure 5 | Reconstruction of contours by Elliptic Fourier descriptors (EFDs). (a) Variation of seed shape that can be accounted for the principal component 1 (PC1) and principal component 2 (PC2). Contours were reconstructed from the corresponding principal component and equal to mean (solid line) or two times the standard deviation (dashed lines). (b) A representative contour of seed shape of respective cultivars reconstructed by the mean values of EFDs coefficients.
